# Practical considerations on hypoxemia and hypoxia in V-V ECMO patients

**DOI:** 10.1186/s13054-024-04972-6

**Published:** 2024-06-24

**Authors:** Dawid L. Staudacher, Matthieu Schmidt, Tobias Wengenmayer

**Affiliations:** 1 Interdisciplinary Medical Intensive Care, Faculty of Medicine and Medical Center, University of Freiburg, Hugstetterstrasse 55, 79106 Freiburg, Germany; 2 1166-ICAN, Institute of Cardiometabolism and Nutrition, APHP, Hôpital Pitié-Salpêtrière, Service de Médecine Intensive-Réanimation, Institut de Cardiologie, Sorbonne Université, Paris, France

We would like to express our gratitude to Aravind Bommiasamy and his coauthors for their detailed analysis on the physiological impacts of cardiac output (CO) on oxygen supply in patients undergoing venovenous extracorporeal membrane oxygenation (V-V ECMO). This foundational knowledge is crucial for effectively managing such complex cases [1].

Understanding the distinctions between hypoxemia and hypoxia is essential for caregivers. Hypoxemia refers to low oxygen saturation in the arterial blood, measurable through indicators such as So2 and paO2. Hypoxia, on the other hand, is the under-supply of oxygen at the tissue level, potentially leading to cell death. Although hypoxemia often results in hypoxia, the relationship is not absolute. For instance, mountaineers at the summit of Mt. Everest might experience significant hypoxemia (paO2 between 19.1 and 29.5 mmHg) without suffering from hypoxia [2]. In contrast, patients who have undergone cardiac arrest may exhibit hyperoxia but still sustain hypoxic brain damage due to compromised cardiac output [3].

At the bedside, while hypoxemia is easily measured, hypoxia is not, making it common practice to treat hypoxemia as a proxy to prevent potential hypoxia, despite hypoxemia not always indicating tissue damage. Therefore, in patients with V-V ECMO experiencing hypoxemia, our intervention algorithm focuses on optimizing oxygen supply. The central strategy here involves managing both the ECMO settings and the patient’s physiological parameters to enhance oxygen delivery. Notably, increasing cardiac output is one of the cornerstones to improving oxygen supply; the use of beta-blockers, which can reduce CO, is contraindicated in these scenarios even if they may appear to improve arterial oxygen saturation [1, 4].

Enhancing CO might initially lead to decreased arterial oxygen saturation, potentially raising concerns among healthcare team members. However, by calculating the product of saturation and CO (with unchanged hemoglobin level) before and after interventions, caregivers can be reassured that the overall oxygen supply to the patient has increased.

Beyond the specific context of VV ECMO therapy, determining the appropriate target values for arterial saturation remains a challenging task, emphasizing the distinct nature of oxygen supply versus saturation in clinical settings (Fig. 1).

**Fig. 1:**
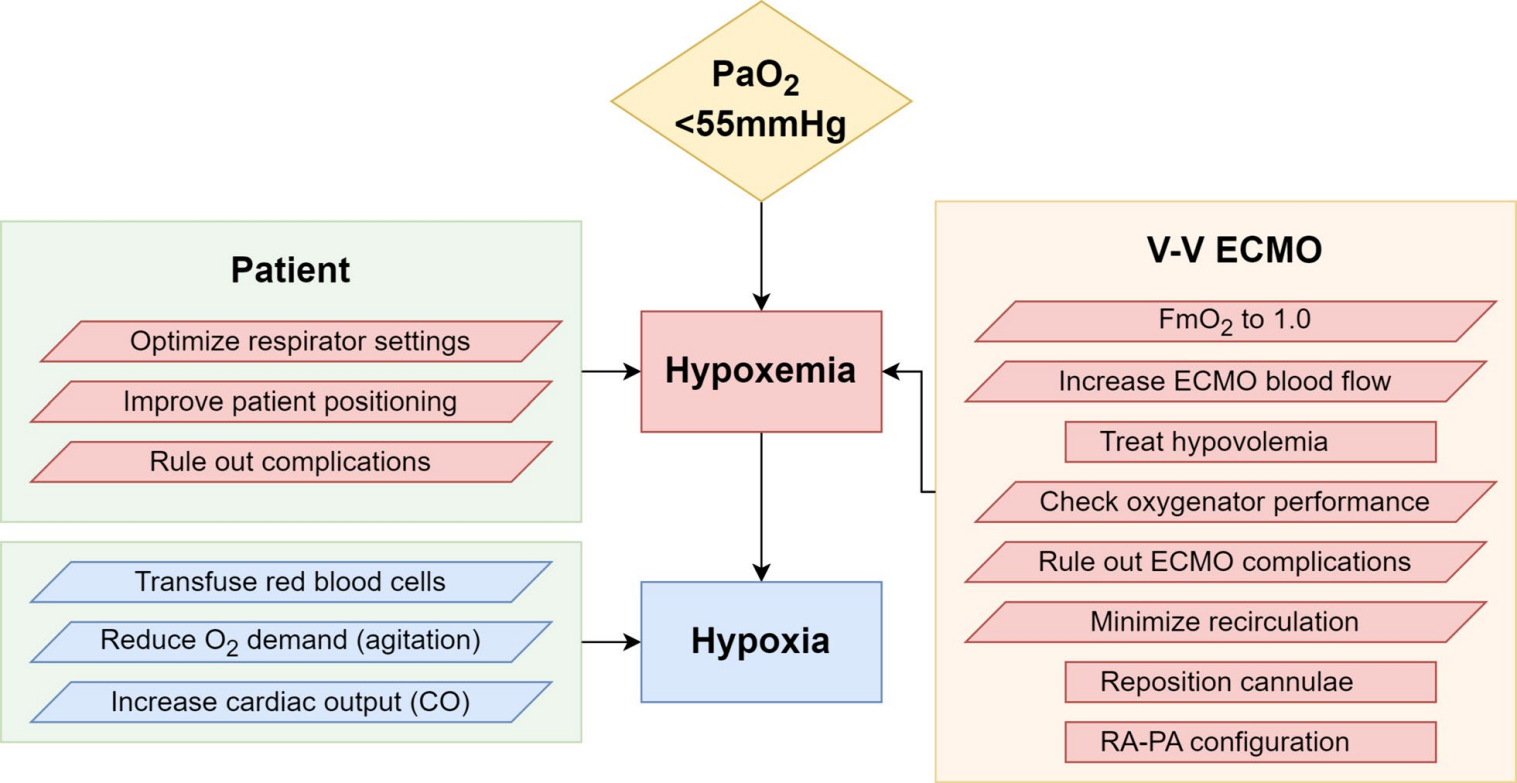
Algorithm to avoid hypoxia in V-V ECMO: Algorithm for preventing and managing hypoxia on V-V ECMO at the bedside. Both, patient as well as V-V ECMO factors must be optimized to improve oxygen supply to the tissue. Note, that increasing CO might lead to lower arterial oxygen saturation but still increase the oxygen supply to the patient. Abbreviations: Fm0_2_: membrane fraction of oxygen, ECMO: extracorporeal membrane oxygenation, RA-PA: right atrium (drainage) to pulmonary artery (return), CO: cardiac output

## Data Availability

Not applicable.
